# MicroRNA‐4286 promotes cell proliferation, migration, and invasion via PTEN regulation of the PI3K/Akt pathway in non‐small cell lung cancer

**DOI:** 10.1002/cam4.2220

**Published:** 2019-05-10

**Authors:** Chunhua Ling, Xueting Wang, Jianjie Zhu, Haicheng Tang, Wenwen Du, Yuanyuan Zeng, Lin Sun, Jian‐An Huang, Zeyi Liu

**Affiliations:** ^1^ Department of Respiratory Medicine the First Affiliated Hospital of Soochow University Suzhou China; ^2^ Suzhou Key Laboratory for Respiratory Diseases Suzhou China; ^3^ Institute of Respiratory Diseases Soochow University Suzhou China

**Keywords:** microRNA, miR‐4286, non‐small cell lung cancer, phosphatase and tensin homologue deleted on chromosome 10, PI3K/AKT signaling

## Abstract

It is well‐known that phosphatase and tensin homologue deleted on chromosome 10 (PTEN) is a tumor suppressor which negatively regulates PI3K/AKT signaling and is activated widely in non‐small cell lung cancers (NSCLC). However, genetic alterations in PTEN genes are rare, suggesting an undefined mechanism(s) for their suppression. Notably, growing evidence indicates that PTEN can be regulated by microRNAs involved in cancer progression. In this study, we discover that the miR‐4286 is overexpressed in NSCLC and negatively regulates the expression of PTEN. Furthermore, we found that miR‐4286 reduces PTEN expression by directly binding to PTEN 3′‐untranslated region (UTR), thereby inhibiting NSCLC cell proliferation and mobility. Moreover, mechanistic investigations revealed that miR‐4286 overexpression was a result of PTEN‐mediated activation of the PI3K/AKT pathway. Taken together, our findings elucidate that miR‐4286 promotes the tumorigenesis of NSCLC by interacting with PTEN. This miR‐4286‐mediated upregulation of PTEN might lead to new therapeutic strategies for NSCLC.

## 1 INTRODUCTION

Lung cancer is the leading cause of cancer‐related death worldwide, and approximately 85% of all lung cancers are non‐small cell lung cancer (NSCLC).^1^, ^2^ Despite the improvements in therapeutic strategies, the outcome of patients with NSCLC remains poor.^3^ Most patients have developed the advanced stage of the disease, which is beyond the optimal treatment period, when the diagnosis is confirmed. Therefore, exploring the pathogenesis of NSCLC remains urgently required, in order to identify new molecular makers and clinical targets.

Growing evidence has shown that there is a close correlation between microRNAs (miRNAs) and development of cancers, including lung cancer.^4^, ^5^ In our previous analysis of miRNA arrays, we found that miR‐4286 level was significantly increased in NSCLC tissues.^6^ It has been reported that miR‐4286 is a promising marker for diagnosing and identifying prognostic markers, including those in esophageal adenocarcinoma^7^, ^8^ and melanoma,^9^ and inhibition of miR‐4286 represses cell proliferation.^10^ But the role of miR‐4286 in NSCLC remains unknown. In current study, we firstly report that miR‐4286 is overexpressed in NSCLC tissues and cell lines and exerts important functions in cell proliferation, migration, and invasion of NSCLC. Meanwhile, we made use of computational algorithms to predict its target mRNAs. It was found that miR‐4286 binds to the 3′‐UTR of phosphatase and tensin homologue deleted on chromosome 10 (PTEN) mRNA. Therefore, miR‐4286 may contribute to the progression of lung cancer cells by regulating the expression of PTEN. As a well‐known tumor suppressor, PTEN can negatively regulate PI3K/AKT signaling and affect normal lung morphogenesis and the prevention of lung carcinogenesis.^11^ A decrease in PTEN expression is a common event and is correlated with poor prognosis in a variety of tumor types. In other cancer types, mutations, chromosomal deletion, and/or Loss of heterozygosity can be frequently observed. Loss of PTEN is also linked with genomic alternations,^12^, ^13^ whereas it is probably attributed to epigenetic silencing in NSCLC.^14^, ^15^ Many studies have reported that PTEN can be regulated by miRNAs involved in cancer progression.^4^, ^16^, ^17^, ^18^, ^19^, ^20^ Here, our study is to assess the mechanism of miR‐4286 in NSCLC tumorigenesis and to find a potential function of miR‐4286 in PTEN downregulation in lung carcinogenesis.

## 2 MATERIALS AND METHODS

### 2.1 Study subjects

From 2009 to 2013, 31 paired frozen NSCLC tissues and adjacent normal lung tissues were obtained from patients in the First Affiliated Hospital of Soochow University. At recruitment, all participants have been offered with the informed consent. Tissue samples were rapidly stored in liquid nitrogen upon collection. Tumor staging was initially determined based on the Revised International System for Staging Lung Cancer. Before sample retrieval， patients were not given chemotherapy or irradiation.

### 2.2 Cell culture

Six NSCLC cell lines (A549, SPC‐A1, H1299, H460, H226, and H1975) were all obtained from the Cell Bank of the Chinese Academy of Sciences (Shanghai, China) and the human bronchial epithelial (HBE) lines were provided by Bogoo Biotechnology (Shanghai, China). The cells were cultured in RPMI 1640 (HyClone, South Logan, UT), supplemented with 10% FBS (Gibco, Carlsbad, CA), 1% L‐glutamine, and 1% penicillin‑streptomycin (Invitrogen, Carlsbad, CA).

### 2.3 Transfection

The oligonucleotide sequences for miR‐4286 mimics, the miR‐4286 inhibitor, and negative controls (NCs) were all purchased by Shanghai GenePharma Company. According to the operator's manual, A549 and H226 cells were plated in 6‐well plates and cell transfection was performed using reagent Lipofectamine 2000 (Life Technologies).

### 2.4 RNA extraction, cDNA synthesis, and quantitative reverse transcription polymerase chain reaction

RNAiso Plus (Takara, Osaka, Japan) was used for extracting total RNA from cell lines and tumor tissues. Reverse transcriptase M‐MLV (Takara) was selected for the synthesis of cDNA. The primers used in reverse transcription and amplification of miR‐4286 and U6 were synthesized by Shanghai GenePharma Company (Shanghai, China).The prime sequence used for miR‐4286 was used according to the following sequence: ACCCCACUCCUGGUACC (forward primer), UACCAGGAGUGGGGUUU (reverse primer).The sequences of reverse transcription quantitative polymerase chain reaction (qRT‐PCR) for PTEN and β‐actin were as follows: PTEN, Forward: 5′‐GGTCTGCCAGCTAAAGGTGA‐3′, Reverse: 5′‐GTTTCCTCTGGTCCTGGTAT‐3′; and β‐actin, Forward: 5′‐CACAGAGCCTCGCCTTTGCC‐3′, Reverse: 5′‐ACCCATGCCCACCATCACG‐3′. Quantitative RT‐PCR reactions were performed using SYBR Premix ExTaq™ (Takara) on an ABI Step One Plus Real‐Time PCR system (Applied Biosystems, Foster City, CA), according to the operator's manual. The expression values of PTEN and miR‐4286 were, respectively, normalized to internal controls β‐actin and U6.

### 2.5 Luciferase reporter assays

Plasmid containing PTEN 3′‐UTR was fused to the 3′ end of psiCHECK2 dual‐luciferase vector (Promega, Madison, WI). The mutated fragments and wild‐type of the PTEN 3ʹ‐UTR containing the predicted miR‐4286 target sites (positions 2969–2975) were synthesized directly (Genewiz, Suzhou, China) and then subcloned into the psiCHECK2 vector to generate thepsiCHECK2‐PTEN‐3′‐UTR wild‐type and a psiCHECK2‐PTEN‐3′‐UTR‐mutant. A549 and H226 cells were plated in a 24‐well plate. The constructed reporter plasmids were co‐transfected with either miR‐4286 mimics or negative control (miR‐NC) into the cells using Lipofectamine 2000 (Life Technologies). Following 48 hours of transfection, the cells were collected and luciferase activity was measured using a Dual‐Luciferase Reporter Assay kit (Promega).

### 2.6 Cell proliferation analysis

Cell proliferation can be measured using the Cell Counting Kit‐8 assay kit (CCK‐8, Boster, Wuhan, China). A549 and H226 cells were seeded in 6‐well plates, and subsequently transfected with miR‐NC, miR‐4286 inhibitors, or miR‐4286 mimics using Lipofectamine 2000. After 48 hours, cells were suspended at 3000 cells per well and then added into 96‐well plates. Ten microliter CCK‐8 was added to each well and incubated for 2 hours at 37°C. The optical density (OD) was measured at 450 nm and cell viability was assessed at 24, 48, and 72 hours separately.

After transfection of miR‐4286 mimics and miR‐4286 inhibitor, cells were seeded into 60‐mm plates and incubated to form colony in culture medium which needs to be refreshed every 3 days. At the end of incubation, 100% methanol and 0.05% crystal violet were used to fix and stain colony, respectively, for 30 minutes. Consequently, the number of macroscopically observable colonies was recorded.

### 2.7 Wound healing, migration, and invasion assays

We made use of wound healing assay to evaluate the ability of cell migration. Cells were plated into 6‐well plates, and after 48 hours transfection, they had formed a monolayer. Gently and slowly scratch the monolayer with a new 10 μL pipette tip across the center of the well, and cells were washed with PBS for two times to remove debris and incubated in fresh medium for an additional 24 hours. The healing process was photographed at 0 and 24 hours with a microscope.

For the migration assay, 4 × 104 cells were added to the upper chamber of Transwell plates (BD Biosciences) in the presence of 1% FBS medium and 10% FBS medium was added as chemoattractant in each lower chamber. Then the cells were allowed to adhere in a 37°C, 5% CO2 incubator for 24 hours. In order to accomplish the invasion assay, 5 × 104 cells in serum‐free medium were added in the upper chamber of an insert precoated with the Matrigel matrix (BD Science, Sparks, MD). The bottom chambers were filled with 10% FBS. After 24 hours, the cells migrated onto the lower surface of the chamber were fixed with 100% methanol and stained with 1% crystal violet. In both assays, cells were photographed and counted under a light microscope.

### 2.8 Cell cycle analysis

The effects of miR‐4286 on the cell cycle of A549 and H226 cells were examined using Cell Cycle Analysis Kit (Beyotime, Shanghai, China) and fluorescence‐activated cell sorting (FACS) Caliber system (Beckman Coulter, Brea, CA). Cells were grown in 6‐well plates and transfected with miR‐NC and miR‐4286 for 48 hours. Then cells were collected and fixed at 4°C in 70% ethanol overnight. Then cells were centrifuged, washed with cold PBS two times, and stained in propidium iodide (PI)/RNase A mixture. Cells were analyzed by flow cytometry after incubation without light for 30 minutes at room temperature.

### 2.9 Western blotting

A549 and H226 cells were transfected with miR‐4286 mimics and miR‐NC or treated with LY294002 (1 µmol/L) 48 hours later. Then, the cells were lysed in RIPA buffer (Cell Signaling Technology, Danvers, MA) with proteinase inhibitor and phosphatase inhibitor cocktail (Sigma‐Aldrich, St. Louis, MO). Protein samples were separated by 10% SDS‐PAGE and transferred to nitrocellulose membranes (Millipore, Billerica, MA). The membranes were incubated with primary antibodies at 4°C overnight with PTEN, AKT, phospho‐AKT (Ser473), cyclin D1 and SNAIL diluted 1:1000 (Cell Signaling Technology, Danvers, MA), and anti‐β‐actin diluted 1:2000 (Santa Cruz Biotechnology, Santa Cruz, CA) and then incubated with the corresponding HRP‐conjugated secondary antibodies for 2 hours at room temperature. Detection was performed using the ECL kit (Pierce, Rockford, IL).

### 2.10 Statistical analysis

All results we obtained are presented as the mean ± standard deviation (SD).Statistical comparisons were determined with Student's *t *test and *P* < 0.05 was regarded as significant. All statistical analyses were performed with GraphPad Prism 5.0 (GraphPad, San Diego, CA) and SPSS 17.0 software (SPSS, Chicago, IL).

## 3 RESULTS

### 3.1 miR‐4286 expression is upregulated in NSCLC tissues and cell lines

**Figure 1:**
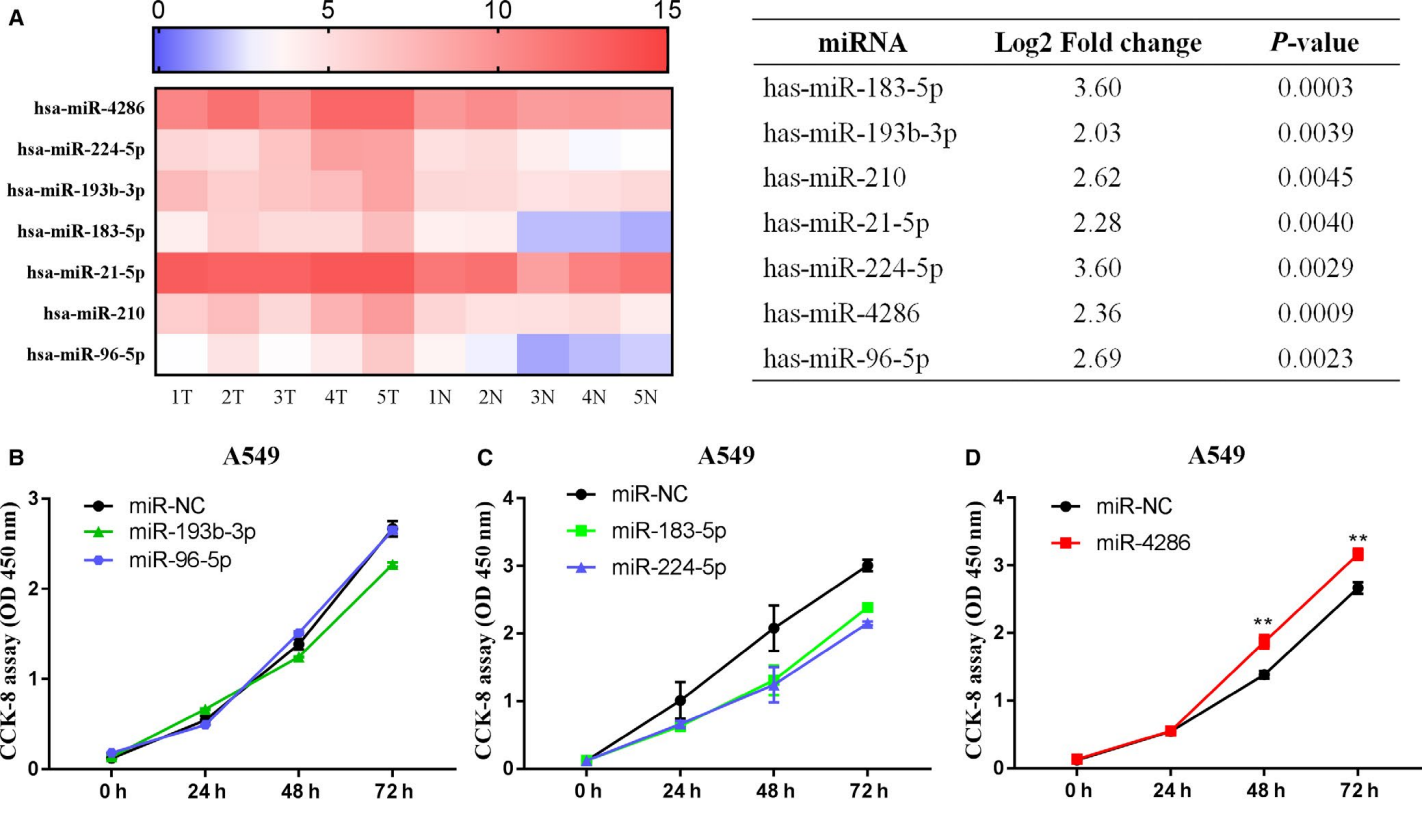
miR‐4286 expression is higher in NSCLC tissues by microRNA array and overexpression of miR‐4286 promotes cell proliferation in A549 cells. (A) MiR‐4286 expression is significantly higher in NSCLC. Log2 fold change >2 and *P* < 0.005. Rows are individual microRNAs; columns are tumor and normal tissue samples. The color scale indicates the relative expression ratio of each miRNA following normalization (red, high expression level; blue, low expression level). (B) The effect of five microRNAs, namely, miR‐193b‐3p, miR‐96‐5p, miR‐183‐5p, miR‐224‐5p, and miR‐4286, in A549 cells. CCK‐8 assay of cell viability in transfected A549 cell lines with five microRNAs mimics at 24th, 48th, and 72th hour; ^**^ *P* < 0.01.

**Figure 2:**
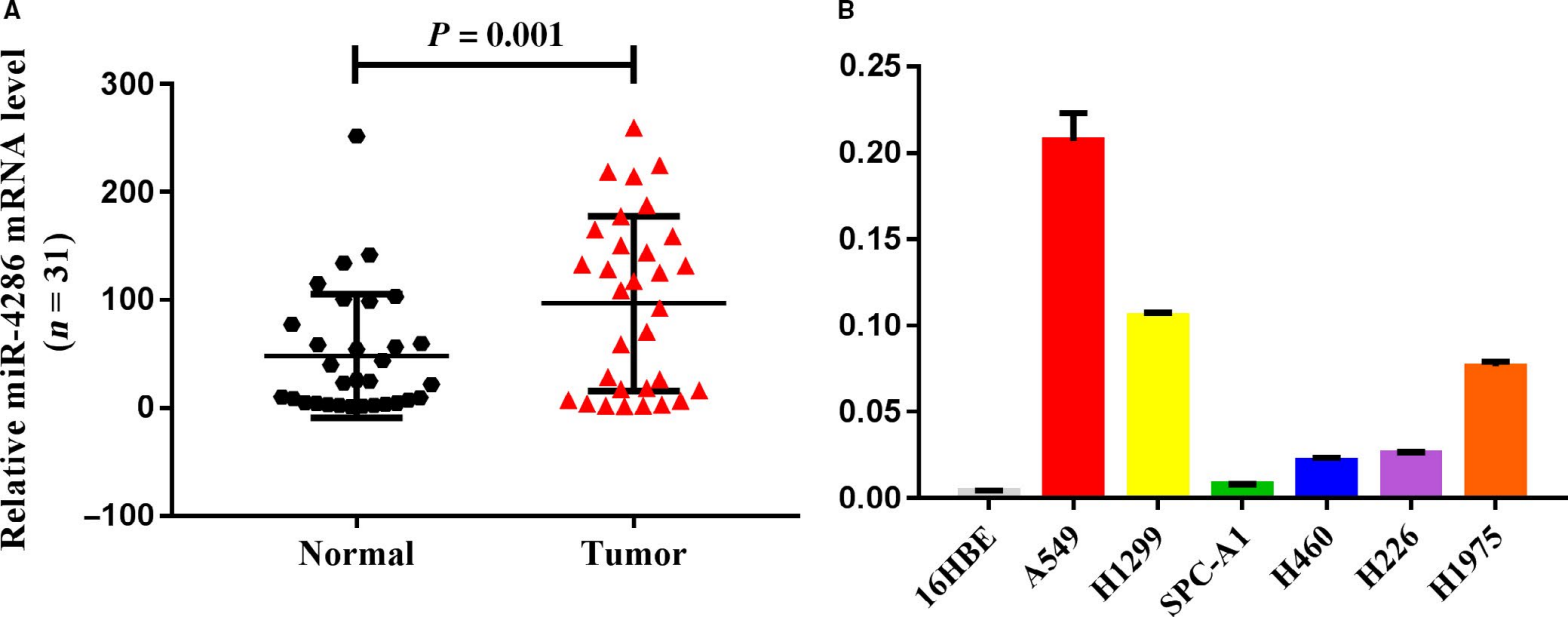
The levels of miR‐4286 are upregualted in human NSCLC tissues and NSCLC cells. (A) MiR‐4286 levels in 31 NSCLC tissues and paired noncancerous lung tissues. (B) MiR‐4286 expression in human NSCLC cell lines and 16HBE; miR‐4286 levels are expressed as a relative index normalized against the expression of U6

MiRNA array data from our previous study showed that 15 miRNA expressions were significantly upregulated in lung carcinoma compared to normal lung tissues.^6^, ^21^More specifically, seven miRNAs (miR‐183‐5p, miR‐193b‐3p, miR‐210, miR‐21‐5p, miR‐224‐5p, miR‐4286, and miR‐96‐5p, log2 fold change ≥2.0, *P* < 0.05) were upregulated (Figure 1A). Among these, miR‐210 and miR‐21‐5p have been reported to be significant diagnostic markers in NSCLC.^22^, ^23^ Therefore, we first explored the function of the five miRNAs (miR‐193b‐3p, miR‐96‐5p, miR‐183‐5p, miR‐224‐5p, and miR‐4286) in the A549 cell line using a CCK‐8 assay. We found that only overexpression of miR‐4286 promoted A549 cell proliferation (Figure 1B‐D, *P* < 0.05). Then, miR‐4286 expression in 31 randomly selected paired NSCLC tissues was significantly upregulated in tumor tissues compared to paired noncancerous tissues (Figure 2A).The expression of miR‐4286 among NSCLC has no relationship with age, gender, smoking status, clinical stage, and lymph node metastasis. Importantly, the results showed that miR‐4286 was significantly associated with histology. (Table 1). Moreover, we found that miR‐4286 expression was upregulated in six NSCLC cell lines than in 16HBE cells (Figure 2B).

**Table 1:** Clinical characteristics and level of miR‐4286 and PTEN mRNA expression in NSCLC tissue

Characteristics	n (%)	miR‐4286 mRNA expression	PTEN mRNA expression
Age			
≤65	20 (65%)	91.72 ± 15.24	0.0438 ± 0.008847
>65	11 (35%)	106.2 ± 31.18	0.06136 ± 0.02409
*P* value		0.6427	0.417
Gender			
Male	17 (55%)	72.11 ± 23.25	0.05606 ± 0.01552
Female	14 (45%)	126.9 ± 12.1	0.04271 ± 0.01259
*P* value		0.0595	0.5218
Histology			
Adenocarcinoma	19 (61%)	128.5 ± 16.92	0.05684 ± 0.01613
Squamous cell carcinoma	8 (26%)	40.17 ± 26.46	0.0474 ± 0.006588
Others	4 (13%)	59.71 ± 27.72	0.02294 ± 0.006263
*P* value		0.019	0.116
Smoking status			
Yes	15 (48%)	80.4 ± 25.64	0.05872 ± 0.01753
No	16 (52%)	112.3 ± 14.54	0.04188 ± 0.01101
*P* value		0.2809	0.4163
Clinical stage			
I	11 (35%)	101.8 ± 24.36	0.03146 ± 0.004367
II	7 (23%)	59.94 ± 25.62	0.03668 ± 0.006255
III	9 (29%)	91.14 ± 29.23	0.05756 ± 0.01896
IV	4 (13%)	160.5 ± 36.52	0.1075 ± 0.06286
*P* value		0.317	0.41
Lymph node metastasis			
No	14 (45%)	104.6 ± 23.7	0.06971 ± 0.02124
Yes	17 (55%)	90.48 ± 18.48	0.03382 ± 0.00375
*P* value		0.6375	0.0779

Data are presented as mean ± SE. An unpaired *t* test was used for two groups. The Kruskal‐Wallis test was used for three or more groups. PTEN, phosphatase and tensin homologue deleted on chromosome 10.

### 3.2 Ectopic overexpression or underexpression of miR‐4286 affects NSCLC cell proliferation via cell cycle progression

**Figure 3:**
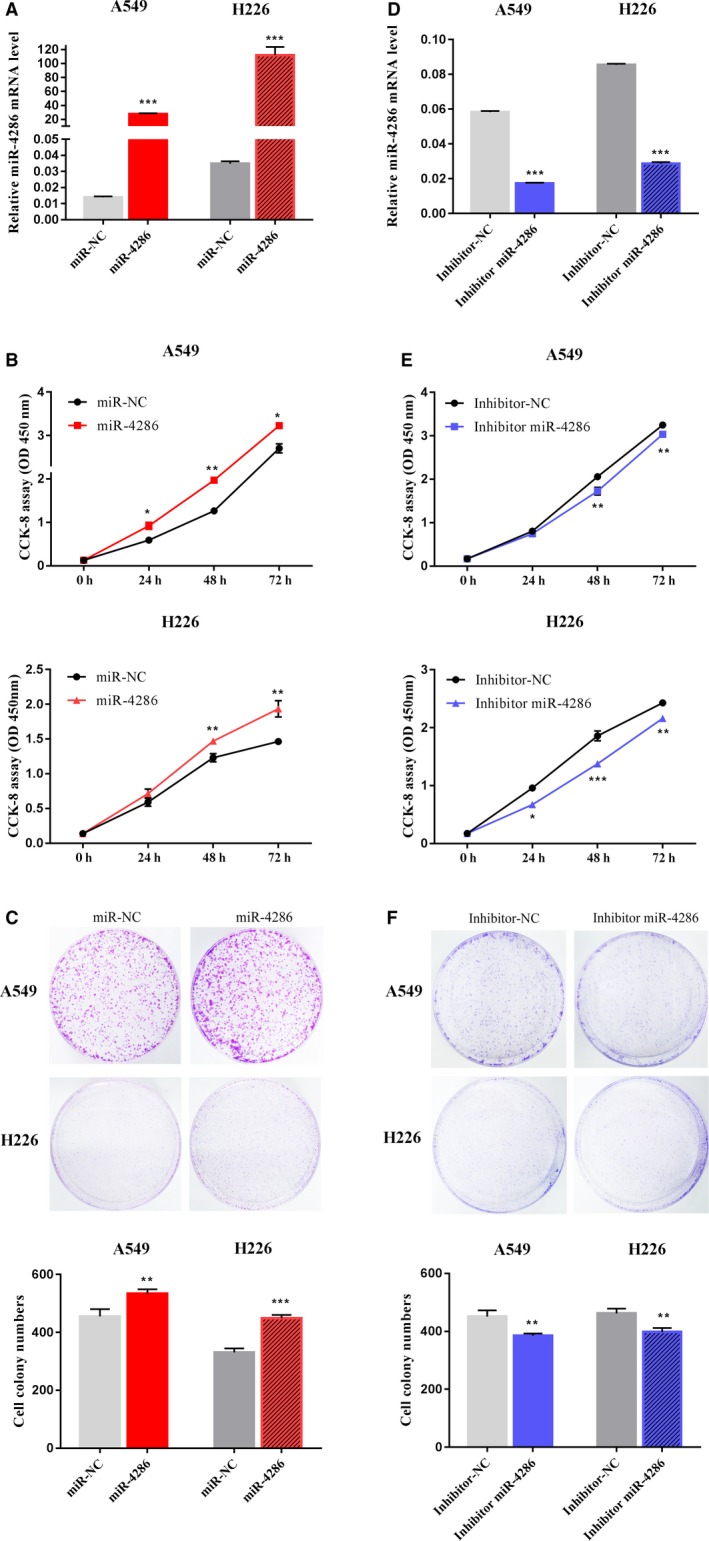
Overexpression of miR‐4286 promotes NSCLC cell proliferation; conversely, underexpression of miR‐4286 inhibits NSCLC cell proliferation. (A) The levels of miR‐4286 in NSCLC cells transfected with miR‐4286 mimics. (B) CCK‐8 assay of cell viability in NSCLC cell lines transfected at hour 24, 48, and 72, respectively, with miR‐4286 mimics (C) Typical images of clonogenic analysis of cell proliferation in NSCLC cells. Bar charts showing clonogenic growth of cells. (D) The levels of miR‐4286 in NSCLC cells transfected with miR‐4286 inhibitor. (E‐F) CCK‐8 assay and clonogenic analysis for cell proliferation in NSCLC cells transfected with miR‐4286 inhibitor

**Figure 4:**
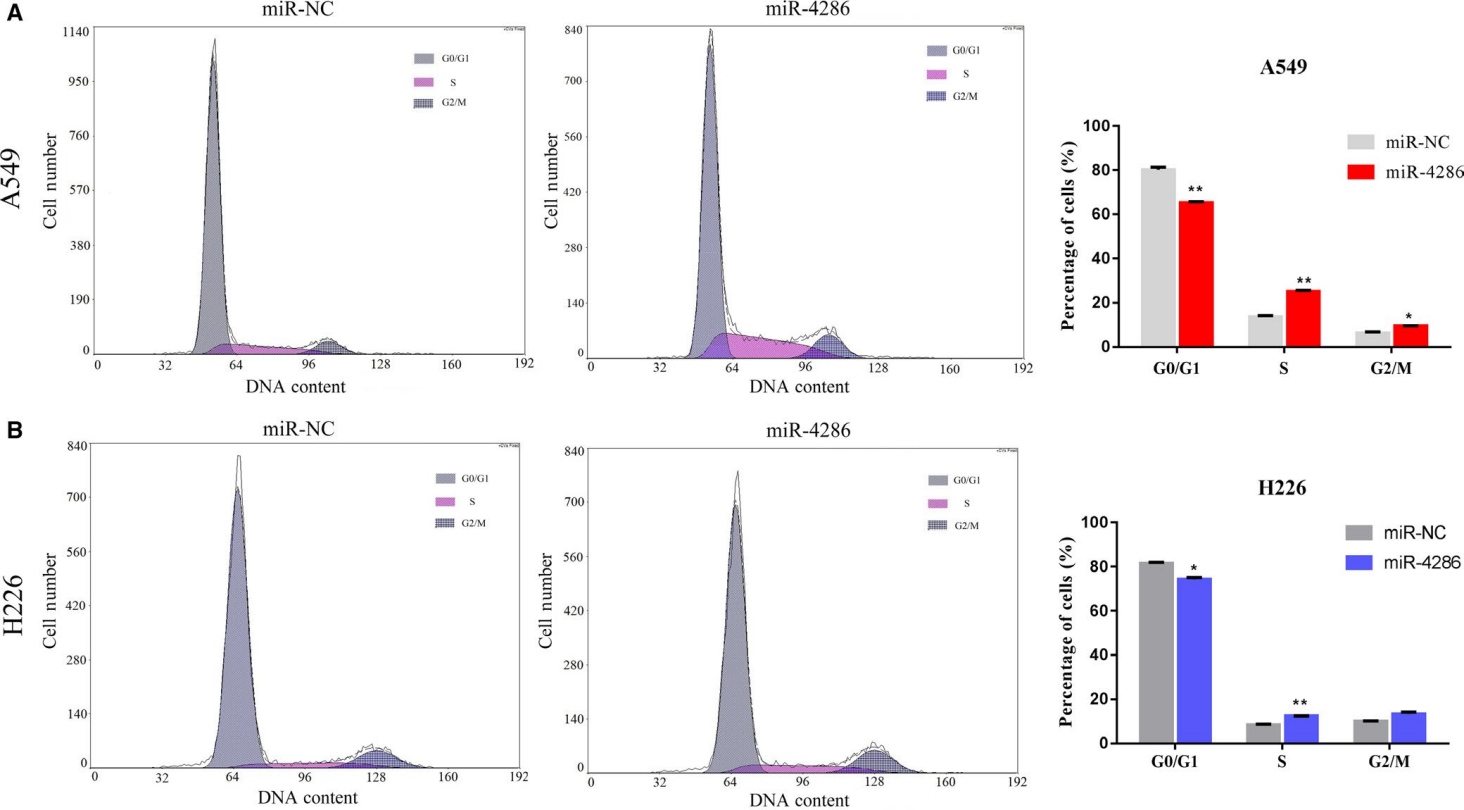
Analysis of NSCLC cell lines by flow cytometry (miR‐4286 vs. miR‐NC cells). Cells were harvested after transfection for 72 hours and then stained with propidium iodide. The proportion of cells for respective periods of cell cycle is shown in the inset of each panel, in which the numbers represent the average value of measurement for three times

We overexpressed miR‐4286 and underexpressed miR‐4286 using miR‐4286 mimics and miR‐4286 inhibitor separately in A549 and H226 cell lines to explore the function of miR‐4286 in NSCLC carcinogenesis. First, the successful overexpression and underexpression of miR‐4286 were confirmed by qRT‐PCR analysis (Figure 3A,D).We used CCK‐8 assays and clonogenic assays to observe the function of miR‐4286 on cell growth. We observed that NSCLC cells transfected with miR‐4286 mimics exhibited significantly accelerated cell proliferation compared with control cells (Figure 3B), and clonogenic assay showed similar results (Figure 3C). By contrast, cell proliferation ability was suppressed by miR‐4286 inhibitor (Figure 3E). These results were also observed by clonogenic assay (Figure 3F).We utilize flow cytometry to analyze the distribution of cell cycle to explore the underlying mechanism of miR‐4286 in promoting cell growth. The percentage of cells in the G0/G1 phase was significantly decreased and meanwhile the proportion of cells in the S phase was significantly increased after transfected with miR‐4286 mimics (Figure 4A,B; *P* < 0.05).

### 3.3 Abnormal expression of miR‐4286 can regulate the migration and invasion abilities of NSCLC cells

**Figure 5:**
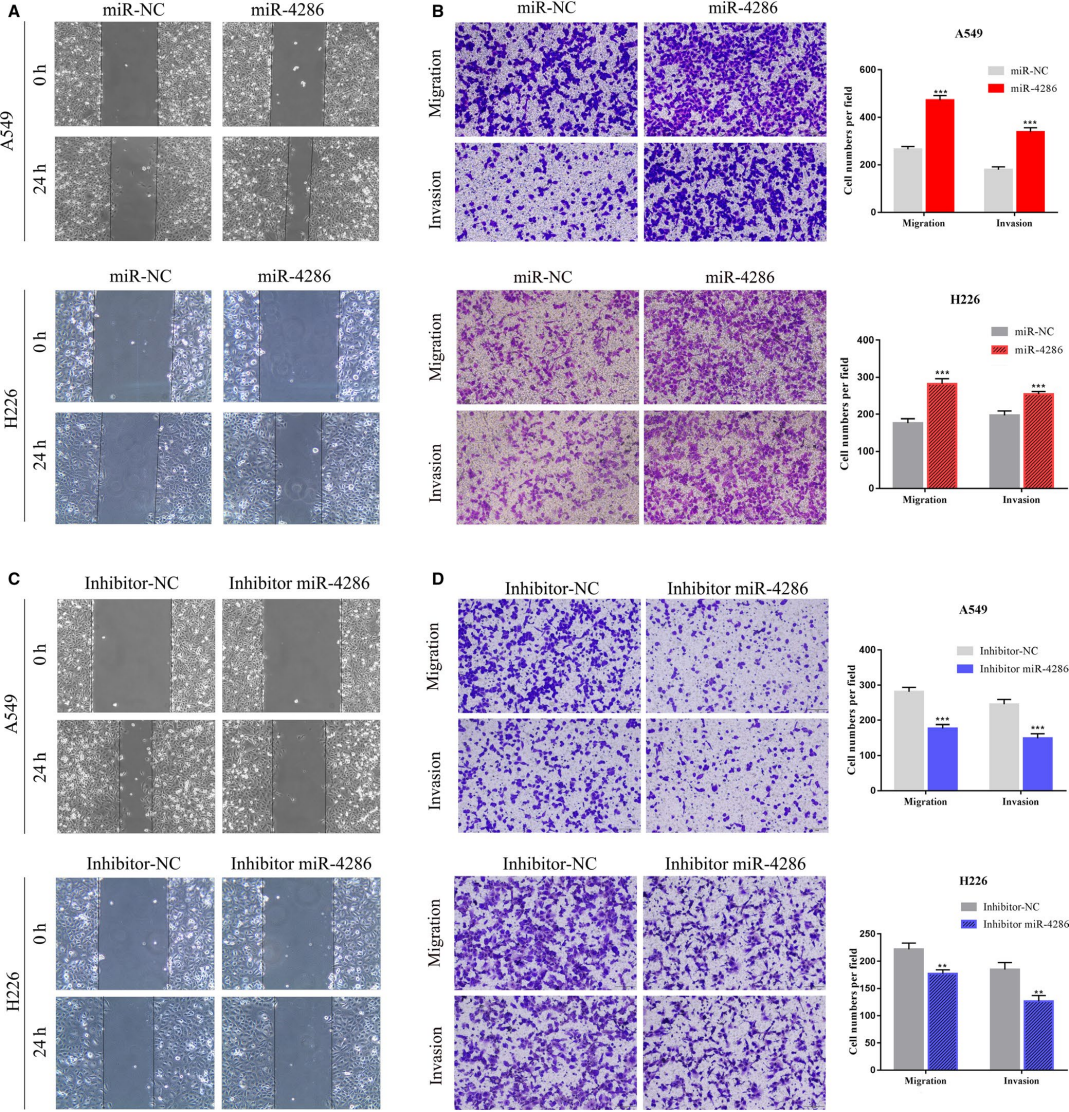
Overexpression of miR‐4286 promotes NSCLC cell migration and invasion; conversely, underexpression of miR‐4286 inhibits NSCLC cell migration and invasion. (A, C) A wound healing assay was conducted to estimate the motility of miR‐4286 transfection in cells. (B, D) Overexpression of miR‐4286 promotes and underexpression of miR‐4286 inhibits not only invasion but also migration of NSCLC cells. Migration of the A549 and H226 cell lines through 8‐μM pore transwells was recorded in the setting of transfection with miR‐4286 mimics or inhibitor. After that, migrated cells were counted in more than three microscopic fields when they were stained (magnification, ×100). Then, cells were treated as described in the front context and designed for invasion through the Matrigel‐coated membrane in the transwells. Cells passing through the membrane were stained and counted with a light microscope. ^**^ *P* < 0.01; ^***^ *P* < 0.001

In addition to function in cell growth, we also assessed the function of miR‐4286 on cell migration and invasion due to either overexpression or underexpression of miR‐4286. We calculated that the influence of miR‐4286 on migration in A549 and H226 cells by wound healing analysis. As shown in Figure 5A, cells transfected with the miR‐4286 mimics showed faster speed toward the scratch than control cells. Similar results were also seen in the transwell assay (Figure 5B). In contrast, miR‐4286 knockdown after transfected with inhibitors significantly decreased the migrated ability of A549 and H226 cells (Figure 5C,D).

### 3.4 PTEN is a target of miR‐4286 through its 3′‐UTR binding in NSCLC cells

**Figure 6:**
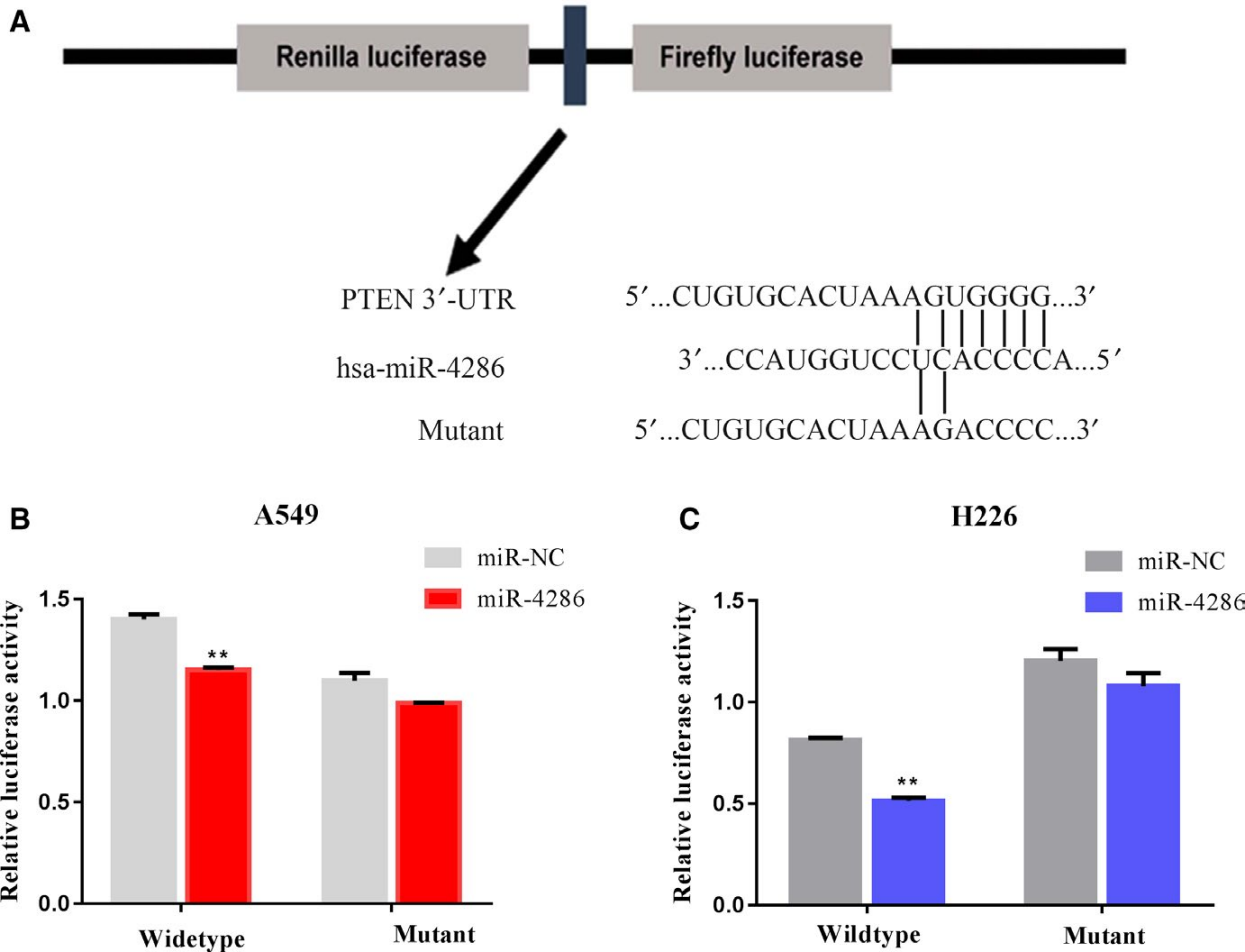
MiR‐4286 reduces *PTEN* expression by directly targeting its 3′‐UTR. (A) Schematic diagram suggesting the subcloning of the miR‐4286 binding site we predicted at position 2969‐2975 of the *PTEN* 3′‐UTR into a psiCHECK‐2 luciferase construct， as well as predicted duplex formation consisting of binding sites of miR‐4286， miR‐4286 wild‐type, or miR‐4286 mutant. (B, C) Luciferase activity of the formation including the wild‐type or mutant *PTEN* 3′‐UTR reporter gene in A549 and H226 cells co‐transfected with the negative control (NC) or miR‐4286. Scrambled sequences were assigned to the NC. Relative Renilla luciferase activity was determined and normalized against the firefly luciferase activity

MiRNAs are critical for attenuating the stability and translation of mRNAs via base pairing to partially complementary sites in the 3′‐UTR of their target genes, and they are involved in multiple physiological and pathological processes. First, through a search of publicly available databases (TargetScanHuman: http://www.targetscan.org/), a complementary sequence of miR‐4286 was identified in the 3'‐UTR of *PTEN* (Figure 6A). To verify this hypothesis, we constructed the PTEN wild‐type (WT) 3'‐UTR (containing the miR‐4286 matching sequence) and PTEN MUT 3'‐UTR (miR‐4286 mutated sequence) plasmids and carried out dual‐luciferase reporter assays in A549 and H226 cells. The data indicated that miR‐4286 suppressed the luciferase activity in PTEN wild‐type group but not in mutant group (Figure 6B,C).

### 3.5 PTEN is frequently downregulated in cell lines and NSCLC tissues

**Figure 7:**
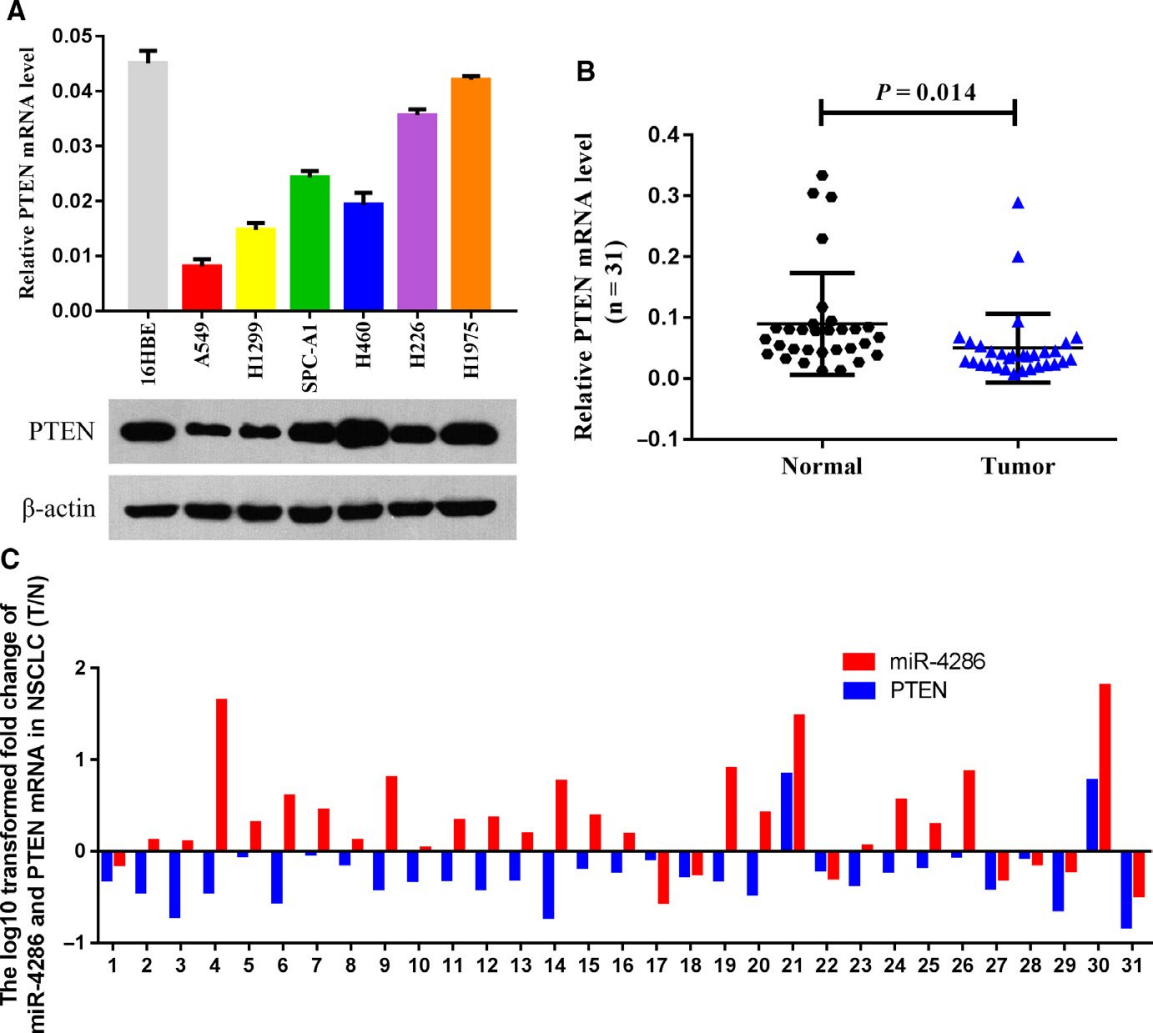
PTEN expression is suppressed in NSCLC cells and tissues and inversely correlated with miR‐4286 overexpression. (A) The level of PTEN in human NSCLC cells was determined by qRT‐PCR and western blotting. (B) The mRNA levels of PTEN in 31 NSCLC tissues versus in normal lung tissues. (C) Corresponding expression of miR‐4286 levels and *PTEN* mRNA in 31 NSCLC tissues. The y‐axis represents the log10 transformed fold change of T/N expression ratios of miR‐4286 levels and PTEN mRNA. The data of each specimen are noted below the x‐axis

First, we determined PTEN mRNA and protein levels in NSCLC and HBE cell lines by qRT‐PCR and western blotting analysis (Figure 7A).The results showed that the PTEN levels were lower in the NSCLC cell lines than in the HBE cell line. Then, we examined 31 pair of NSCLC tissues and adjacent normal lung tissues and found that *PTEN* mRNA expression was downregulated in NSCLC tissues (Figure 7B). Considering the small sample size, there was no significant association between miR‐4286 expression in NSCLC tissues and clinical characteristics including age, gender, smoking status, clinical stage, histology, and lymph node metastasis (Table 1).However, after further analysis, we found that 21 NSCLC tissues (67.74%) with a high miR‐4286 level also presented low expression of PTEN mRNA (Figure 7C). Collectively, our data show that PTEN is remarkably downregulated in NSCLC tissues and cell lines.

### 3.6 PI3K/AKT signaling contributes to the miR‐4286‐mediated malignant phenotype of NSCLC cells

**Figure 8:**
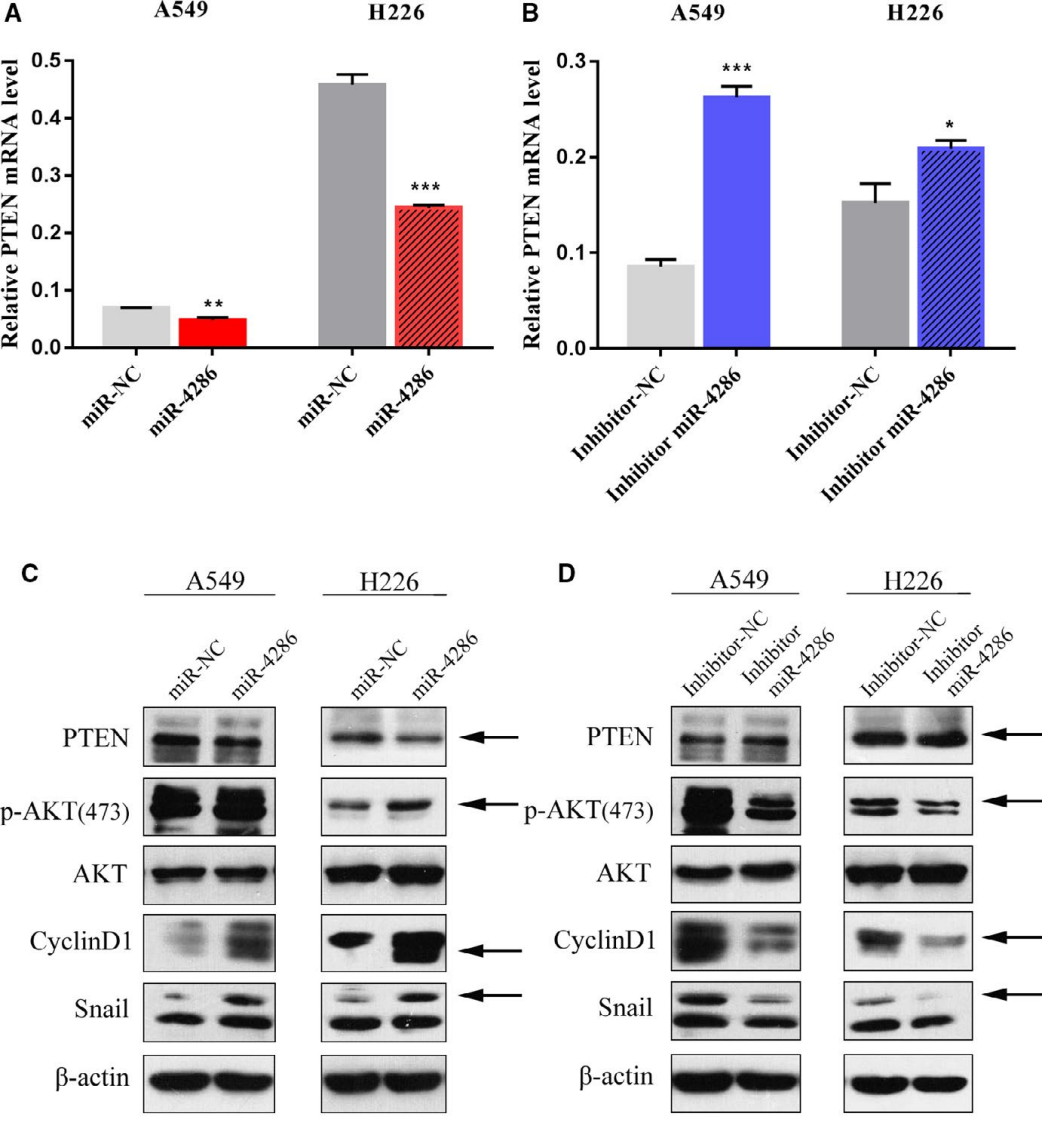
PI3K/AKT signaling contributes to the miR‐4286‐mediated malignant phenotype of NSCLC cells via PTEN. (A, B) PTEN mRNA expression in NSCLC cells transfected with miR‐4286 mimics or inhibitor and the control cells transfected with miR‐NC. (C, D) The A549 and H226 cells were treated with miR‐4286 mimics/the miR‐4286 suppressor or not for 72 h, respectively. The expression levels of p‐AKT, AKT, cyclin D1, and Snail were analyzed by western blotting. ^*^ *P* < 0.05; ^**^ *P* < 0.01; ^***^ *P* < 0.001

PTEN functions as a well‐known tumor suppressor, which negatively regulates PI3K/AKT signaling. First, we induced overexpression or underexpression of miR‐4286 using miR‐4286 mimics or inhibitors and then determined the level of PTEN in cells (Figure 8A,B). Notably, ectopic miR‐4286 expression using mimics in A549 and H226 cells remarkably decreased PTEN expression, displaying enhanced phosphorylation of AKT (Figure 8C), whereas knockdown of miR‐4286 using inhibitors in A549 and H226 cells robustly suppressed phosphorylation of AKT (Figure 8D).Our results indicated that miR‐4286 indeed activated the PI3K/AKT pathway.

## 4 DISCUSSION

Recently, several studies have profiled miRNA expression directly in lung cancers, and unique groups of miRNAs have been identified that either characterize neoplastic tissues or identify patients with poor prognosis. 6, 19, 26 Our previous study indicated that miR‐4286 is significantly increased in NSCLC based on miRNA arrays,^6^ and similar results were observed in esophageal carcinoma and melanoma.^7^, ^8^, ^9^ A recent study suggested that overexpression of miR‐4286 may promote the development of esophageal carcinoma through JAK2/STAT3 pathway activation by targeting INPP4A. 27 However, the mechanism of miR‐4286 in NSCLC remains unknown. In this study, we first explored the function of miR‐4286 in NSCLC and found that ectopic overexpression or underexpression of miR‐4286 is involved in NSCLC cell proliferation, colony formation, invasion, and migration. Our results revealed that miR‐4286 may function as an oncogene in NSCLC development.

Loss of PTEN expression is observed in a variety of tumor types, including lung cancer. However, the mechanism by which PTEN expression is regulated in NSCLC remains unclear. Although mutation of PTEN is an infrequent event, PTEN protein expression is frequently decreased or absent in lung cancer.^12^ Recent evidence suggests that miRNAs are related with the regulation of PTEN in cancer progression,^13^, ^14^, ^15^, ^16^, ^17^ including lung cancer. 4, 28, 29, 30, 31 Furthermore, several studies suggest that miRNA‐mediated downregulation of PTEN might lead to new therapeutic strategies for NSCLC. 32, 33, 34 In this study, using bioinformatics and luciferase assays, we first found that PTEN is a target gene of miR‐4286. Then, we examined the mRNA expression of PTEN in NSCLC tissues and normal lung tissues from 31 NSCLC patients and found that 21 NSCLC tissues (67.74%) had a high miR‐4286 level while presenting low PTEN mRNA expression (Figure 7C). Moreover, the expression of miR‐4286 in tissues from NSCLC patients with lymph node metastasis was significantly higher than that in tissues from NSCLC patients without lymph node metastasis. Therefore, our findings revealed the mechanistic interaction between miR‐4286 and PTEN in NSCLC carcinogenesis.

PTEN is a well‐known tumor suppressor that negatively regulates PI3K/AKT signaling in lung cancer. Therefore, we induced overexpression or underexpression of miR‐4286 in NSCLC cells to examine the effects of miR‐4286 on the PTEN/PI3K/AKT axis. Our results indicated that miR‐4286 indeed activated the PI3K/AKT pathway. In conclusion, this is the first time for us to confirm that miR‐4286 expression is upregulated in NSCLC and related with PTEN expression negatively. What's more, we found that miR‐4286 functions as oncogene in NSCLC by directly targeting 3'‐UTR of PTEN to attenuate the expression of PTEN, repressing NSCLC cell proliferation and mobility. So, the results from our study provide evidence on the association between miR‐4286 and PTEN in NSCLC carcinogenesis. This miR‐4286‐mediated upregulation of PTEN might provide a novel approach for the treatment of NSCLC.

## CONFLICT OF INTERESTS

The authors declare that they have no conflict of interest.

## AUTHOR CONTRIBUTION

X.‐T.W. and Z.‐Y.L. designed the study. C.‐H.L., X.‐T.W., J.‐J.Z., H.‐C.T., and W.‐W.D. collated the data, designed, and developed the database, Y.‐Y.Z. and L.S. carried out data analyses and preparation of figures. C.‐H.L., J.‐A.H., and Z.‐Y.L. contributed to drafting and revising the manuscript. All authors have read and approved the final submitted manuscript.
